# Renal Abscess and Recurrent Bacteremia Caused by Campylobacter Infection in an Adult With Common Variable Immunodeficiency

**DOI:** 10.7759/cureus.21827

**Published:** 2022-02-02

**Authors:** Joana Cameira, Patrícia Araújo, Ana Afonso, Daniel Oliveira, Filipa Ceia

**Affiliations:** 1 Internal Medicine, Unidade Local de Saúde do Alto Minho, Viana do Castelo, PRT; 2 Infectious Diseases, Centro Hospitalar Universitário São João, Porto, PRT

**Keywords:** secondary prophylactic antibiotherapy, common variable immunodeficiency, renal abscess, campylobacter extraintestinal manifestations, recurrent campylobacter infection

## Abstract

*Campylobacter* bacteremia and extraintestinal manifestations are extremely rare, occurring more frequently in immunocompromised patients. We report a rare clinical case of a 46-year-old female with common variable immunodeficiency (CVID) presenting with a previously undescribed extraintestinal complication - renal abscess - and recurrent bacteremia caused by *Campylobacter*, which was a therapeutic challenge and required the use of secondary prophylactic antibiotic treatment to prevent recurrence.

## Introduction

*Campylobacter jejuni* infection is among the most frequent causes of diarrhea in both industrialized and developing countries [1]. In humans, *Campylobacter* is mainly responsible for gastroenteritis, reported in 7.5% of patients with common variable immunodeficiency (CVID), the most frequent primary antibody deficiency (PAD) [2]. Gastrointestinal complications can occur due to the direct spread of the infection to the adnexal organs, resulting in cholecystitis, pancreatitis, and peritonitis [3]. Extraintestinal manifestations, in turn, are extremely rare and include meningitis, endocarditis, septic arthritis, osteomyelitis, and sepsis. Bacteremia is detected in less than 1% of patients, occurring more frequently in immunocompromised patients, in which the bacterial translocation process is favored, facilitating the passage of bacteria from the intestinal mucosa to the bloodstream [1]. In patients with PAD, bacteremia and extradigestive localizations are more frequent (25% versus 0.15%-2% and 22% versus 7%, respectively) [4,5], and the recurrence rate is higher (42%) compared with 1.2% in the general population [4,6]. This may lead to repeated hospitalizations and impaired quality of life. Despite frequent hospitalizations, the overall prognosis is good, as described in one series where, in 45 patients with 97 episodes of *Campylobacter* infection, one death occurred [4]. In this study, it is suggested that reinfection is more likely than persistent colonization since the molecular profiles of strains from patients with recurrent infections were all different, although colonization with multiple strains cannot be excluded [4]. *Campylobacter* infection in patients with CVID also tends to be more resistant to therapy when compared to healthy individuals [4,7]. We report the clinical case of an immunocompromised female with a previously undescribed complication of *Campylobacter* *jejuni* infection - renal abscess - and recurrent *Campylobacter* bacteremia that is difficult to manage, with the need of multiple antibiotic treatments, human IV immunoglobulin (IVIg) adjustments, and secondary prophylactic antibiotherapy to prevent recurrence.

## Case presentation

We describe a clinical case of a 46-year-old female presenting with recurrent fever caused by *Campylobacter *infection. Her past medical history included a CVID diagnosis at the age of 19, and she was under monthly treatment with 30 g of IVIg, usually with serum immunoglobulin G (IgG) levels of 500-600 mg/dL (normal range: 700-1600 mg/dL) before administration, immunoglobulin M (IgM) < 5 mg/dL (normal range: 40-230 mg/dL), and immunoglobulin A (IgA) < 5 mg/dL (normal range: 60-400 mg/dL). She also had splenectomy at the age of 26 due to immune thrombocytopenia, pulmonary hypertension at the age of 44, and chronic liver disease due to right heart failure.

The first episode of *Campylobacter* infection occurred at the age of 44 when the patient was diagnosed with gastroenteritis, and the first culture isolation of *Campylobacter jejuni* in feces was made. She was medicated with 500 mg azithromycin for five days. Previously and after this episode, self-limited episodes of diarrhea were described but without identification of *Campylobacter* or other infectious agents.

**Figure 1:**
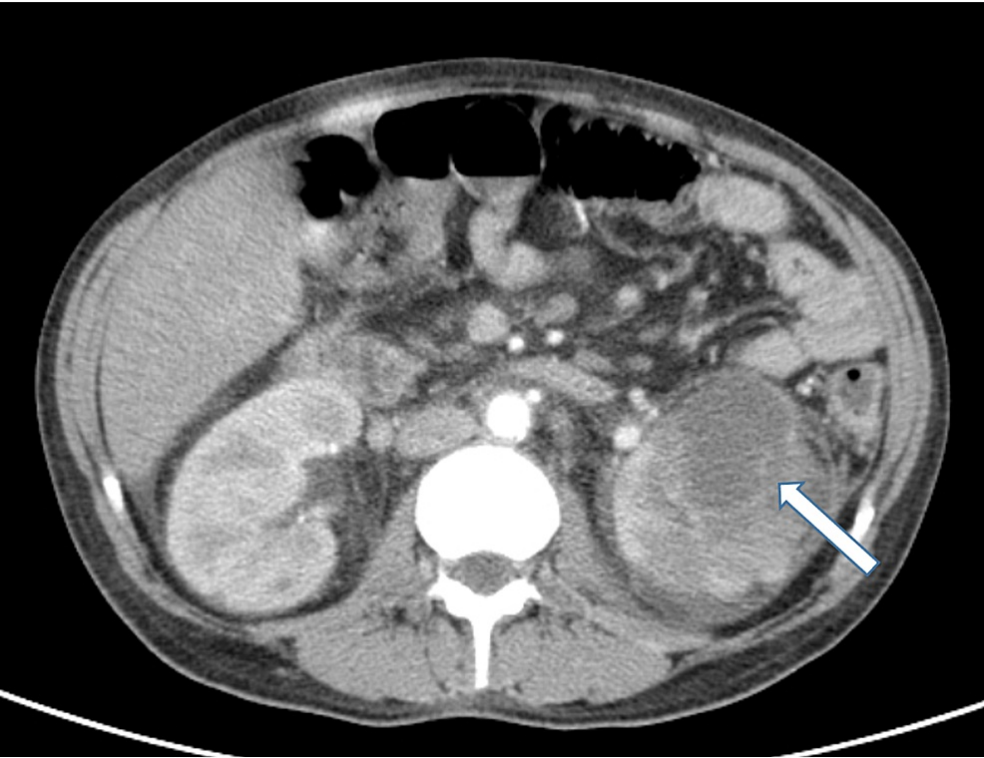
Abdominopelvic CT scan showing the presence of renal abscesses, 7 cm on the left (white arrow) and 3 cm on the right CT: computed tomography

Two years after the first *Campylobacter jejuni *gastroenteritis, the patient was admitted to the emergency department with fever and lower extremity edema. Two weeks before, two-day self-limited diarrhea was also described. On admission, she was hemodynamically stable and apyretic and had a painless abdomen with medium volume ascites and symmetrical edema of the lower limbs. The analytical study revealed leukocytosis (21.17 × 10^9^/L) with 76.6% neutrophils, C-reactive protein (CRP) of 4.36 mg/dL, altered liver parameters (alkaline phosphatase: 908 UI/L; gamma-glutamyl transferase: 187 UI/L), and normal renal function. Chest radiography and venous Doppler of the lower limbs did not show alterations. Abdominal ultrasound showed heterogeneous hepatomegaly and moderate to large volume ascites; no biliary duct distention, kidney cysts, or obstructive nephropathy were described. Ascitic fluid was slightly turbid, with 2542 cells, approximately 1500 neutrophils (60%). The albumin serum-ascites gradient was 2.2. Newly diagnosed ascites and spontaneous bacterial peritonitis were identified in the patient with chronic liver disease, and antibiotic therapy with ceftriaxone was started. Stool, blood, and ascitic fluid cultures were negative. On the sixth day of ceftriaxone, the patient had a recurrence of fever. An abdominopelvic computed tomography (CT) scan was performed, which showed the presence of bilateral renal abscesses, 7 cm on the left, loculated, and 3 cm on the right (Figure 1).

**Figure 2:**
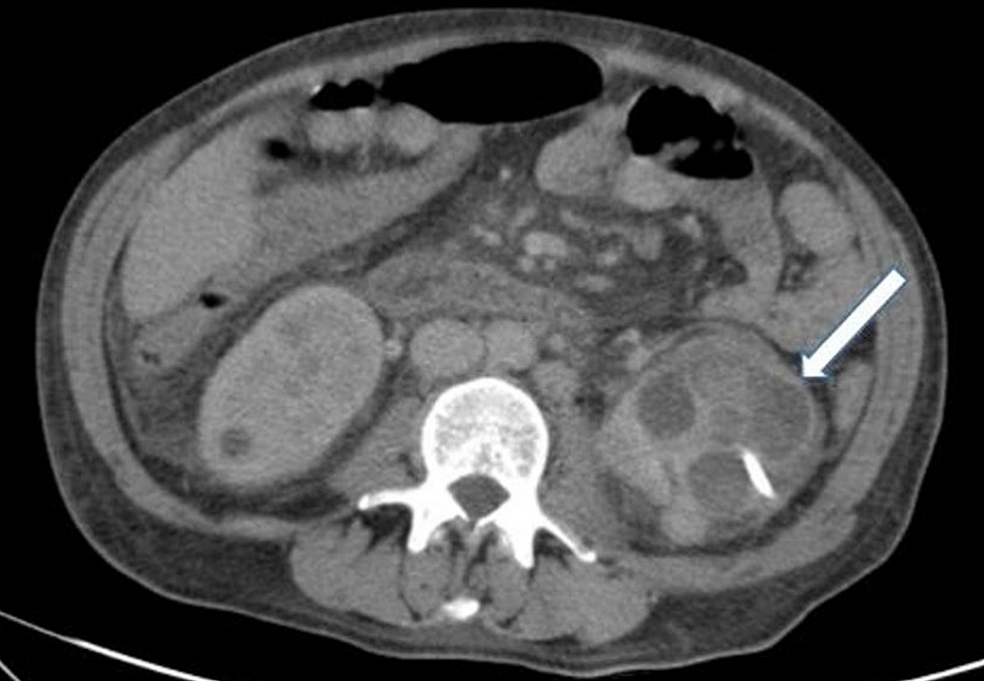
Abdominopelvic CT scan showing the reduction in the right abscess and significant increase on the left, with the presence of new abscesses (white arrow) CT: computed tomography

Antibiotic therapy was maintained, and a percutaneous drain was placed in the left renal abscess. New blood and pus specimens were collected. On the 14th day of ceftriaxone, there was a fever recurrence, and the CT scan showed a reduction in the right abscess but a significant increase on the left with the presence of new abscesses (Figure 2). It was therefore decided to change the antibiotic therapy to piperacillin/tazobactam, new drainage of the left renal abscess was performed, and a new dose of 35 g of IVIg (three weeks after the last dose) was administered to the patient due to the severe infection and low IgG levels (245 mg/dL).

**Figure 3:**
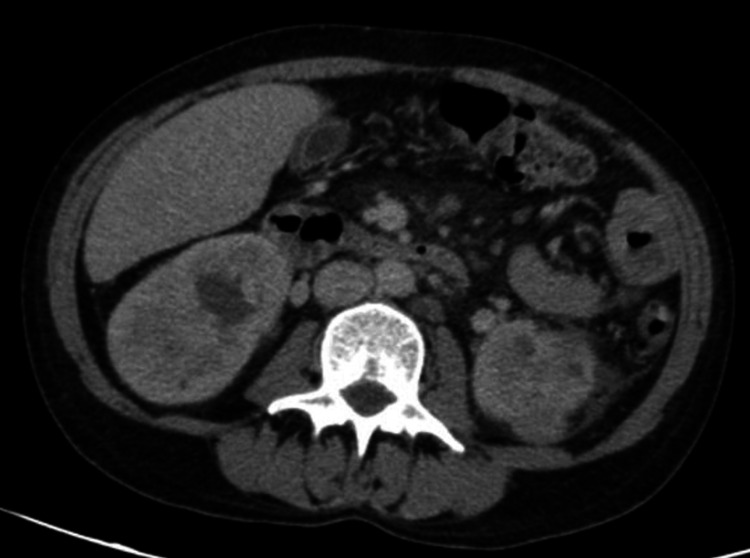
Abdominopelvic CT scan showing no renal abscess recurrence CT: computed tomography

Since then, the patient had clinical improvement with sustained apyrexia and lowering of inflammatory parameters. Three weeks after culture incubation, *Campylobacter jejuni* was identified in two blood cultures and in the pus of the drained abscess (no antibiogram available). Antibiotic therapy was changed to IV ciprofloxacin plus oral azithromycin due to severe infection with clinical, analytical, and imaging improvement. The patient was discharged to complete six weeks of antibiotic therapy with oral ciprofloxacin 500 mg twice daily and azithromycin 500 mg once daily. In the fourth week of outpatient antibiotic therapy, the patient developed fever and watery stools. Blood work showed leukocytosis and CRP increase, and CT scan showed no renal abscess recurrence (Figure 3). The patient was admitted with suspicion of *Campylobacter* infection recurrence; therefore, hospitalization was decided for IV antibiotic therapy with imipenem and gentamicin, with improvement. This time, blood cultures identified *Campylobacter coli*, and an antibiogram was solicited, which showed sensitivity to carbapenems and tetracycline, and resistance to ciprofloxacin, piperacillin/tazobactam, and amoxicillin/clavulanic acid. The control blood culture performed after eight days of hospitalization had no growth, the fecal stool culture was negative, and the colonoscopy showed no changes. After 14 days of antibiotic therapy, the patient was discharged. Of note, our hospital laboratory does not have the capacity to perform *Campylobacter* antibiograms, which demanded an external laboratory examination with the consequent delay in the final results.

One month after the first *Campylobacter* bacteremia recurrence, the patient presented in the outpatient clinic with fever, an increase in inflammatory parameters, and low IgG levels (276 mg/dL). Blood cultures were collected, with the growth of *Campylobacter coli*. Antibiogram continued to show sensitivity only to tetracyclines and carbapenems. The patient was admitted, and after a multidisciplinary discussion, antibiotic therapy was started with meropenem and gentamicin, IVIg administration interval was shortened to two to three weeks, and the dose was increased to 35 g. Positron emission tomography (PET) showed no metastatic infection. Since it was considered *Campylobacter* infection recurrence, after three weeks of antibiotic therapy, the patient underwent intestinal eradication for *Campylobacter* with paromomycin for eight days, at a dose of 500 mg three times a day. The patient was informed about the importance of taking precautions to prevent exposition to *Campylobacter*, such as avoiding ingestion of raw poultry meat, raw and unproperly washed vegetables, use of proton pump inhibitors, and other measures. After infection treatment, IgG levels remained within the normal range. The new interval and dose of IVIg were maintained.

Six months after the second recurrence, new *Campylobacter jejuni* bacteremia and low IgG levels (355 mg/dL) were identified. Imipenem and doxycycline were started, and after three weeks of antibiotic therapy and one extra administration of IVIg, the patient was discharged medicated with oral doxycycline 100 mg twice daily as secondary prophylaxis; however, after two weeks, the patient suspended medication without medical indication.

Four months after the third recurrence, *Campylobacter jejuni* was identified again, and an antibiogram showed, similar to the previous *C. coli *antibiogram, only sensitivity to carbapenems and tetracycline. Meropenem and doxycycline were started for three weeks. It was explained again to the patient the importance of chronic medication with doxycycline. Since then, the patient has been taking doxycycline continuously without recurrence of campylobacter infection for 24 months, IgG levels remained within the normal range, and it was possible to increase the interval of IVIg each three to four weeks.

## Discussion

*Campylobacter* infection is generally self-limited, and no treatment is required. However, in immunocompromised hosts, severe infections with prolonged, complicated, and recurrent bacteremia may occur [4,7], as seen in our case. We herein describe the first case of renal abscess due to *Campylobacter jejuni*. Bacteremia spreading seems to be the most probable cause of the renal abscess, and we can only speculate about a possible mechanism of translocation from intestinal mucosa-ascites-kidney. It is important to note that prior to the first bacteremia episode and even in the first blood cultures collected, we cannot exclude an episode of bacteremia since, without the clinical suspicion, the blood cultures were managed in a conventional mode.

In primary hypogammaglobulinemia, *Campylobacter* bacteremia often requires antibiotic therapy and immunoglobulin replacement, which reinforces the importance of humoral immunity mechanism in combating *Campylobacter* infection [4,7]. Immunoglobulin M and, more specifically, immunoglobulin A play an important role in mucosal immunity, contributing to the clearance of *Campylobacter* from the intestinal tract. Studies report that *Campylobacter* enteritis occurred more frequently in patients with CVID with undetectable serum IgA levels compared with patients with normal IgA levels [4,7]. In this case, there were several recurrences of bacteremia despite the optimization of IVIg therapy, which can be explained by the low concentration of IgA and IgM in the IVIg batch. However, the optimization of IVIg was crucial since IgG is also very important to neutralize the bacteria as shown by the clear relationship between active infection and IgG levels lowering due to its consumption. In our case report, bacteremia recurrence was caused by two different *Campylobacter* - *C. jejuni *and* C. coli* - a finding that probably represents reinfection rather than a chronic carriage as referred to in the literature [4], although a chronic carriage certainly cannot be excluded.

The antibiotics commonly used to treat *Campylobacter* infection include macrolides, fluoroquinolones, aminoglycosides, tetracyclines, and beta-lactams [8-10]. Less than 1% of *C. jejuni* and 10% of *C. coli *are resistant to macrolides, which are the first-line treatment [11]. Fluoroquinolones can also be a treatment option; however, resistance to these is increasing, which limits their effectiveness (almost 50% of *C. jejuni* and 60% of *C. coli*,* *with studies reporting up to 90% resistance in humans) [9,11]. In cases of strains resistant to quinolones and macrolides, or disease progression, amoxicillin with clavulanic acid or carbapenems should be used, depending on the severity of the case [9,11]. *Campylobacter* species are inherently resistant to trimethoprim and some beta-lactams (penicillin and cephalosporins); therefore, these should be avoided [12]. In the case of chronic manifestations of *Campylobacter* infection and if macrolide resistance is verified, the use of the combination of amoxicillin and clavulanic acid or tetracyclines and fluoroquinolones has been suggested [11].

This case highlights a therapeutic challenge due to several episodes of *Campylobacter* bacteremia recurrence in which multiple antibiotic schemes were used and resistance to the instituted antibiotic therapies were confirmed. The fact that antibiotic sensitivity tests to *Campylobacter* were not routinely performed in our laboratory was also a difficulty, which prevented the rapid adequacy of antibiotic therapy.

Due to persistent recurrence despite adequate therapy (antibiotics and IVIg optimization), other therapeutic solutions needed to be addressed. Considering the absence of IgA, which favors translocation of *Campylobacter* in the intestinal mucosa, the decision to initiate intestinal eradication of *Campylobacter* with paromomycin was made, however without success. Running out of options, although antibiotic prophylaxis is not recommended [1], based on a case report [13] and as a desperate measure, secondary antibiotic prophylaxis was initiated with doxycycline 100 mg every 12 hours with success, and there was no recurrence of bacteremia after 24 months of follow-up since her last episode.

## Conclusions

We report the first case of *Campylobacter jejuni* renal abscess, and we also focus on difficult management of recurrent *Campylobacter* infection in a patient with CVID. We believe that after adequate antibiotic therapy, IVIg adjustment, and correction of environmental factors, if recurrent *Campylobacter* infection persists, secondary antibiotic prophylaxis with doxycycline, adapted to antibiogram, may be a therapeutic option as a last resort for patients without complete response. However, further research is needed to confirm the effectiveness of these regimens.
